# The White Operating-Theatre

**Published:** 1919-09-27

**Authors:** 


### THE WHITE OPERATING-THEATRE.

We have read during the past few years in both the English and American Press a number of articles advocating the use of tinted walls or wainscoting, dark-coloured accessories, or a general suppression of the "dazzling" whiteness of our operating-theatres. More recently we see a vigorous defence of the white theatre by an American surgeon, who, from the technical side of the question at least, puts forward a very strong case indeed. We can leave aside the sundry possible causes and secondary interests which may have made journals ever ready for constructional innovation or for finding uses for wares recently placed on the market, and must decide the question from the standpoint of the surgeon himself, who should undoubtedly be the person best qualified to judge.

One of the first considerations to the operator—the man upon whom success must largely depend—is the question of glare and eye-strain. The Americans, always strong in surgical technique, seem to be unanimous in declaring that glasses of special tinting and construction to eliminate the irritating violet rays and treated in one of the many ways which prevent steaming are both efficient, comfortable, and allow of full light concentration on the wound. The complications of artificial lighting and absence of shadow formation can be worked out to the full if those immediately concerned at the site of operation are thus protected and in comfort. It is unnecessary here to discuss the relative merits of diffuse and central illumination—both have their claims under different surgical circumstances and with proper equipment can be provided at will, regardless of local colouring. It is probable that whatever tinting were used, to the surgeon himself and his personal work very little difference would be made.

There are, however, quite other advantages in a general whiteness. The assistants and theatre nurses require long training to attain the perfect "aseptic conscience," and we believe that the pure white surfaces act as constant, often necessary, reminders towards scrupulous care. Again, there is, when anæsthesia must be induced in the actual theatre or more particularly in the case of regional anæsthesia, the psychic effect on the patient. Possibly habit and custom have produced lay approval of the white theatre, but white has been the popular symbol for cleanliness from time immemorial, and may thus produce a sub-conscious effect inherited through the ages. The cleaning services of our hospitals are relatively good, but it is obvious that dirt, splashes of blood or infected fluids are more easily detected on a white surface, and thorough though an orderly service may be, the surer we are of cleanliness the better. The whole question is one of efficiency, and we believe that the greatest and best work with the minimum effort can be performed by surgeons, sisters, and orderlies amid the white surfaces to which we are now so well accustomed.

